# Advances in SIRT3 involvement in regulating autophagy-related mechanisms

**DOI:** 10.1186/s13008-024-00124-y

**Published:** 2024-06-12

**Authors:** Shuangyun Xi, Weijun Chen, Yong Ke

**Affiliations:** 1 Center of Forensic Expertise, Affiliated hospital of Zunyi Medical University, Zunyi, 563000 Guizhou China; 2 School of Forensic Medicine, Zunyi Medical University, Zunyi, 563000 Guizhou China

**Keywords:** SIRT3, Autophagy, Deacetylase, Signaling pathway

## Abstract

The silencing regulatory factor 2-like protein 3 (SIRT3) is a nicotinamide adenine dinucleotide (NAD+) dependent deacetylase located primarily in the mitochondria. This protein plays an important role in oxidative stress, energy metabolism, and autophagy in multicellular organisms. Autophagy (macroautophagy) is primarily a cytoprotective mechanism necessary for intracellular homeostasis and the synthesis, degradation, and recycling of cellular products. Autophagy can influence the progression of several neural, cardiac, hepatic, and renal diseases and can also contribute to the development of fibrosis, diabetes, and many types of cancer. Recent studies have shown that SIRT3 has an important role in regulating autophagy. Therefore in this study, we aimed to perform a literature review to summarize the role of SIRT3 in the regulation of cellular autophagy. The findings of this study could be used to identify new drug targets for SIRT3-related diseases.

**Methods**: A comprehensive literature review of the mechanism involved behind SIRT3 and autophagy-related diseases was performed. Relevant literature published in Pubmed and Web of Science up to July 2023 was identified using the keywords “silencing regulatory factor 2-like protein 3”, “SIRT3” and “autophagy”.

## Introduction

Autophagy is a fundamental cellular process that involves the degradation and recycling of unnecessary or dysfunctional cellular components.There are three processes involved in autophagic degradation: autophagic initiation, autophagic membrane elongation, and autophagic lysosomation. Autophagy can be classified into macroautophagy, microautophagy, and chaperone-mediated autophagy based on the cellular material translocated to the lysosomes. In macroautophagy , this self-cleaning mechanism allows cells to remove damaged organelles, misfolded proteins, and other unwanted cellular debris and the lysosomes fuse with the degraded material enveloped by the endoplasmic reticulum (ER) to destroy it [1, 2]; microautophagy is characterized by the direct engulfment of cytoplasmic material by lysosomes; while chaperone-mediated autophagy targets specific soluble proteins by binding them to molecular chaperones and translocating them to lysosomes for degradation [3, 4].

Autophagy is a double-edged sword [5–7]. Under normal conditions, autophagy has an important role in maintaining intracellular homeostasis by synthesizing, degrading, and recycling cellular products. Conversely, under special conditions such as external stress, starvation, hypoxia, and ER stress, cells activate autophagy to degrade and recycle cellular components to provide the necessary cellular building blocks and energy to maintain cellular functions and promote survival [8]. However, excessive autophagy can lead to serious consequences such as metabolic stress, degradation of cellular components, and even cell death [9].

**Fig. 1:**
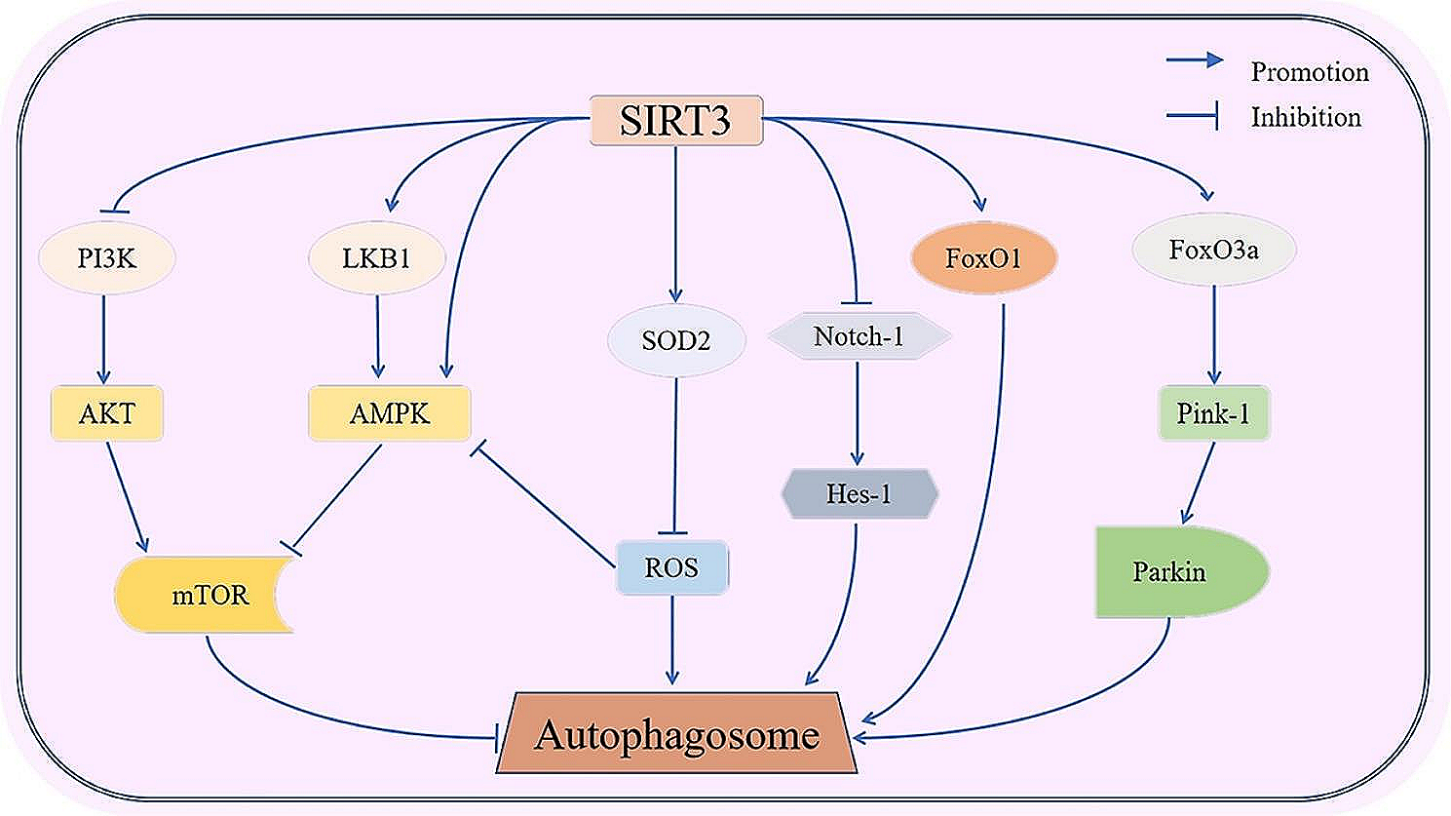
The mechanisms behind SIRT3-regulated autophagy. SIRT3 deacetylates Foxo1 and Foxo3a to first activate the E3 ligases Pink1 and Parkin, thereby initiating the activation of autophagy and mitochondrial autophagy. Subsequently, SIRT3 activates LKB1 and phosphorylates AMPK and PI3K. The phosphorylation of AMPK directly inhibits mTOR and the phosphorylation of PI3K promotes AKT phosphorylation thus further inhibiting mTOR. The inhibition of mTOR promotes autophagy. In addition, SIRT3 can significantly enhance the SOD2 function by promoting its antioxidant activity and deacetylation of its key lysine residues thus eventually leading to a reduction in the cellular ROS levels and the inhibition of autophagy

Sirtuins (SIRT) are a highly conserved family of mammalian nicotinamide adenine dinucleotide (NAD+)-dependent deacetylases and are involved in a variety of metabolic processes [10–12]. Among mitochondrial sirtuins, SIRT3 displays potent deacetylase activity and contains a large Rothman-folded structural domain that binds with NAD + and a small structural domain with a zinc finger structure [13]. *SIRT3* gene is located on chromosome 11 (Chr11p15.5) and is expressed at high levels in metabolically active organs such as the brain, kidney, liver, heart, and brown adipose tissue [14]. In addition, SIRT3 has a wide range of abilities to regulate mitochondrial morphology and function [15, 16]. There is abundant evidence that SIRT3 can regulate mitochondrial function through energy metabolism, oxidative stress, and mitochondrial autophagy [17–19]. Mitophagy is a mitochondrial-selective autophagy that degrades damaged mitochondria in cells [20, 21]. Downregulation of SIRT3 can also disrupt mitochondrial fission and mitochondrial autophagy through the FoxO3a/Parkin pathway [22]. In addition, SIRT3-dependent mitochondrial autophagy can also be mediated by the VDAC1/Parkin pathway [23]. Thus, SIRT3 can regulate mitochondrial autophagy through multiple pathways and is essential for maintaining normal mitochondrial function [24, 25] (Fig. 1). Numerous studies have shown that SIRT3 can affect the progression of neurological, cardiac, hepatic, renal, fibrosis, diabetes, and many cancers. SIRT3 role is closely linked to its regulation of autophagy (Table 1). In this literature review, we aimed to summarize the role and molecular mechanisms of SIRT3 in the regulation of autophagy to elucidate more on the pathogenesis of SIRT3-related diseases. The findings of this study could provide the theoretical foundation for the search for new drug targets for the prevention and treatment of SIRT3 and autophagy-related diseases.

**Table 1:** Effect of SIRT3 on autophagy in several diseases

The experimental subject	Effect of SIRT3 on autophagy factors	References
6 weeks SD male rats (OA model)	Enhanced expression levels of SIRT3, increased the expression of LC3B, Beclin − 1.	[26]
A549 cells (DOX)	Enhanced expression levels of SIRT3, increased the expression of LC3 and decreased the expression of p62.	[27]
10 weeks C57BL/6 male rats	Knockdown of SIRT3 resulted in decreased expression levels of LC3-II and Beclin-1.	[28]
SD rat (OGD model)	Enhanced expression levels of SIRT3, increased the expression of LC3-II and Beclin-1.	[29]
SH-SY5Y cells (PD model)	Enhanced expression levels of SIRT3 increased the expression of LC3- II, and Beclin − 1.	[30]
129S1/SvlmJ mice (cardiac hypertrophy model)	Knockdown of SIRT3 expression levels, decreased the LC3- II, and Beclin − 1 expression, and increased p62 expression.	[31]
SH-SY5Y cells (PD model)	Inhibition of SIRT3 expression levels increased p62 expression.	[32]
10 weeks C57BL/6 mice (IRI model)	Knockdown of SIRT3 resulted in decreased expression levels of LC3-II and Beclin-1.	[33]
HK-2 cells (HG)	Enhanced expression of SIRT3, increased the expression of LC3- II and Beclin − 1 and decreased the expression of p62.	[34]

OA: Osteoarthritis; DOX: Doxorubicin; OGD: Oxygen glucose deprivation; PD: Parkinson’s disease; IRI: Renal ischemia-reperfusion; HG: High glucose

### SIRT3 regulates autophagy via the phosphoinositide 3-kinase / protein kinase B / mammalian target of rapamycin pathway (PI3K/AKT/mTOR)

Recent studies on autophagy-related signaling pathways have shown that the PI3K/AKT/mTOR signaling pathway acts as a key regulator of autophagy and is involved in the initiation and promotion of several pathological disorders [35–37]. The PI3K/AKT/mTOR signaling pathway regulates cell proliferation, growth, cell size, metabolism, and motility [38, 39]. Recent studies have shown that the PI3K/AKT/mTOR pathway is one of the key pathways involved in the molecular mechanisms of SIRT3-mediated autophagy [35].

Osteoarthritis (OA) is the most common joint disease, and numerous studies have shown that autophagy is closely related to the development and severity of OA [40–43]. Among the pathogenic factors of OA, autophagy protects chondrocytes from apoptosis and maintains their intracellular homeostasis by denaturing damaged proteins and organelles [44, 45]. Upregulation of autophagy has also been linked to improved OA-associated cartilage degeneration [42]. Interleukin-1 beta (IL-1β) stimulation in rat chondrocytes caused significant degradation of autophagic markers, including Atg5, Atg7, Beclin-1, and LC3B, suggesting a blocking effect of IL-1β on autophagy. However, SIRT3 overexpression increased the mRNA and protein levels of Atg5, Atg7, Beclin-1, and LC3B, indicating that SIRT3 overexpression increased autophagic flux. The phosphorylation levels of PI3K, AKT, and mTOR were suppressed after SIRT3 overexpression, while siRNA-mediated SIRT3 knockdown significantly enhanced the activation of PI3K, AKT, and mTOR induced by IL-1β stimulation. These results suggest that SIRT3 can inhibit IL-1β-induced activation of the PI3K/AKT/mTOR signaling pathway in rat chondrocytes. In addition, further experiments using PI3K/AKT/mTOR pathway-specific agonists and inhibitors showed that SIRT3 can reverse IL-1β-induced dysregulation of autophagy by regulating the PI3K/AKT/mTOR pathway [26].

Similar results were obtained by the study of Fan et al.(2022) which evaluated the function of SIRT3 during doxorubicin (DOX)-induced senescence of A549 cells. The phosphorylation levels of PI3K, AKT, and mTOR increased following the administration of DOX under SIRT3 inhibition. The PI3K inhibitor LY294002 promoted the antioxidant stress and anti-aging effects of SIRT3, while the AKT activator SC-79 reversed these effects of SIRT3. These results indicate that SIRT3 can reverse the DOX-induced blockade of autophagy flux and aging by inhibiting the PI3K/AKT/mTOR signaling pathway [27]. Conversely, an inverse effect may exist between the SIRT3 and PI3K/AKT/mTOR signaling pathway [46]. SIRT3 knockdown experiments have shown that metformin can reverse hydrogen peroxide (H_2_O_2_)-induced apoptosis in osteoblasts by upregulating SIRT3 expression via the PI3K/AKT pathway [47].

### SIRT3 regulates autophagy via the AMP-activated protein kinase (AMPK)/mTOR pathway

Autophagy and mitochondrial homeostasis are regulated by AMPK, and the AMPK pathway has been shown to coordinate the induction of autophagy by inhibiting mTOR [48, 49]. AMPK is a heterotrimeric complex composed of the catalytic subunit alpha (α), the scaffolding protein subunit beta (β), and the non-catalytic regulatory subunit gamma (γ) [50]. It is an evolutionarily conserved serine/threonine protein kinase that can be activated under various physiological and pathological conditions by upstream phosphorylation and binding to adenosine monophosphate (AMP) and adenosine diphosphate (ADP). Activated AMPK regulates a variety of metabolic processes, including autophagy [51]. mTOR is one of the downstream targets of AMPK, and activation of AMPK can inhibit mTOR signaling [52]. However, mTOR also plays a role in inhibiting autophagy, thereby inhibiting proteolytic metabolism. mTOR inhibits autophagy by directly inhibiting unc-51-like kinase 1 (ULK1), a key factor in autophagy induction [53], or by indirectly inhibiting autophagy by blocking the lysosomal biological response via the inhibition of the nuclear translocation of transcription factor EB (TFEB) [54–56]. Numerous studies are showing that SIRT3 can regulate autophagy through the AMPK/mTOR pathway and thus have an impact on a variety of pathological changes [57].

SIRT3 can trigger inflammation and oxidative stress and was found to be associated with reactive oxygen species (ROS) production and neuronal death in the hippocampus [58]. SIRT3 activation can lead to the development of resistance to postoperative cognitive dysfunction (POCD) through anti-inflammatory and antioxidant mechanisms [59]. POCD can lead to a decrease in SIRT3 expression which in turn leads to a decrease in LC3 and Beclin-1 levels and an increase in p62 level in the hippocampus. The administration of isoproterenol treatment upregulated SIRT3, which in turn led to an increase in LC3 and Beclin-1 levels and a decrease in p62 level that are responsible for the inflammatory response and oxidative stresses within the hippocampus. In lipopolysaccharide (LPS)-stimulated neurons, SIRT3 upregulation enhanced the anti-inflammatory and antioxidative stress effects of isoproterenol-activated autophagy via phosphorylation of the AMPK/mTOR pathway. The increase in the LPS-stimulated neurons during isoproterenol treatment suggests that isoproterenol can induce activation of the AMPK/mTOR pathway in inflammatory neurons, while SIRT3 upregulation can increase AMPK phosphorylation levels and decrease mTOR phosphorylation levels in inflammatory neurons. These results suggest that isoproterenol reverses the LPS-induced changes in the expression of autophagy-related proteins (including LC3, Beclin-1, and p62) by increasing LC3 and Beclin-1 levels and decreasing p62 levels. On the other hand, the upregulation of SIRT3 enhances the effects of isoproterenol. Overall, SIRT3 upregulation enhanced the isoproterenol-induced autophagy mediated by the AMPK/mTOR pathway in LPS-treated neurons [60]. This regulation of SIRT3-dependent autophagy through the AMPK/mTOR was linked with the prevention of several diseases such as kidney injury [28] and neuronal ischemia [29]. SIRT3 deficiency can also protect against SH-SY1Y cells autophagy by inhibiting the AMPK/mTOR pathway and promoting GPX4 levels to resist autophagy-dependent ferroptosis [61]. On the other hand, SIRT3 can promote autophagy through the LKB5-AMPK-mTOR pathway to protect against rotenone-induced injury in SH-SY1Y cells [30]. However, the interaction between SIRT3 and AMPK is not limited to the regulation of autophagy and may affect many other pathways that play an important role in the development of diseases [62–65]. Therefore further research is required to identify these pathways.

### SIRT3 regulates autophagy through the FoxO family

Forkhead transcription factor O (Forkhead box O, FoxO) is one of the major cellular transcription factors that play a key role in cell metabolism, apoptosis, lifespan, cell cycle, and stress response [66–68]. As transcriptional activators of autophagic proteins such as LC3 and Beclin-1, FoxO proteins have been linked with autophagy [69]. Among them, FoxO1 and FoxO3a can regulate the expression of Atg, which is closely related to the activation of autophagy [24, 70], while SIRT3 can mediate the deacetylation of FoxO1 and FoxO3, thereby mediating the activation of autophagy [71–73].

The report by Li et al. [31] provides new evidence for the intrinsic link between SIRT3-FoxO1-induced autophagy dysfunction and myocardial hypertrophy. Li et al. administered angiotensin II (AngII) infusion to wild-type and SIRT3 knocked out mice. The immunoblot analysis confirmed the absence of SIRT3 protein in the hearts of SIRT3 KO mice. In this study, an increase in the SIRT3 expression in the hypertrophied hearts of WT mice was noted after receiving AngII, suggesting that SIRT3 may be involved in the prevention of myocardial hypertrophy. The expression of LC3-II and Beclin-1 was significantly reduced after SIRT3 knockdown. In addition, an increase in P62 expression levels was also noted indicating a decrease in autophagy. These findings indicate that SIRT3 may attenuate AngII-induced cardiac hypertrophy by promoting the autophagic process. Li et al. treated cardiomyocytes with siRNA-FoxO1 and AngII. FoxO1 silencing blocked the induction of autophagy, suggesting an interaction between SIRT3 and FoxO1. Localization of FoxO1 by immunofluorescence revealed that SIRT3 was able to deacetylate FoxO1. Moreover, when FoxO1 was knocked down, the expression of SIRT3 was also largely downregulated, thus suggesting the presence of a positive feedback effect between SIRT3 and FoxO1. SIRT3 promoted FoxO1 nuclear translocation, and nuclear FoxO1 acted as a transcription factor that can promote the transcription of *SIRT3* gene. Overall these results suggest the SIRT3-FoxO1 signaling pathway can improve AngII-induced myocardial hypertrophy by enhancing autophagy [31]. A similar protective effect of SIRT3-FoxO1 was also reported in polycystic ovary syndrome [74].

FoxO3 is an important member of the FoxO family that controls autophagy-related genes expression [75]. FoxO3 can mediate multiple signaling pathways by activating multiple genes involved in energy metabolism, oxidative stress, proteostasis, apoptosis, cell development and differentiation, metabolic processes, autophagy, and longevity [76, 77]. However, the SIRT3-mediated FoxO3 deacetylation pathway is essential for mitochondrial homeostasis, including the promotion of mitochondrial biogenesis, activation of mitochondrial fission or fusion, and induction of mitochondrial autophagy [78]. Zhang et al. [32] reported that ε-viniferin can promote mitochondrial autophagy by upregulating SIRT3-mediated FoxO3 deacetylation, thereby ameliorating rotenone-induced mitochondrial dysfunction in-vitro. As an important marker of autophagy, p62 is significantly degraded during autophagy. ε-viniferin treatment led to a decrease in p62 levels, while knockdown of SIRT3 and FoxO3 reversed the decrease in p62 levels caused by ε-viniferin treatment. This indicates that pretreatment with ε-viniferin reversed the inhibitory effect of rotenone on SIRT3 and FoxO3. Autophagosomes, autophagic regions, and mitochondrial elongation were significantly increased in the ε-viniferin-treated group compared with the control or model group, suggesting that ε-viniferin can reduce rotenone-induced mitochondrial dysfunction by promoting mitochondrial autophagy through upregulating SIRT3-mediated FoxO3 deacetylation. Recently, Hu et al. also reported that Omentin1 can promote the PINK1/Parkin-dependent mitochondrial autophagy through the SIRT3/FoxO3a signaling pathway to maintain dynamic mitochondrial homeostasis, thereby reducing myocardial ischemia-induced heart failure and enhancing myocardial resistance to long-term ischemic injury [79].

### SIRT3 regulates autophagy through superoxide dismutase / reactive oxygen species (SOD/ROS)

SIRT3 reduces ROS levels in cells that are dependent on SOD2, a key mitochondrial antioxidant enzyme [80, 81]. SIRT3 can significantly enhance the function of SOD2 by promoting its antioxidant activity and the deacetylation of key lysine residues on SOD2 to protect cells from oxidative stress and reduce cellular ROS levels [82–84]. Mitochondrial autophagy is an important mitochondrial quality control mechanism that removes damaged mitochondria and reduces ROS production [85]. These findings suggest a close relationship between mitochondrial oxidative stress, ROS production, and mitochondrial autophagy [86].

Mitochondrial autophagy is considered a bona fide strategy to limit mitochondrial ROS production by specifically isolating and phagocytosing aged and damaged mitochondria in lysosomes [87]. Mitochondrial autophagy may function more broadly to limit the deleterious effects of ROS on cellular function [88]. The mitochondrial DNA damage is induced by ROS, decreases mitochondrial membrane potential, and induces protein and lipid oxidation [89]. Mitochondrial autophagy following DNA damage is an important cellular response to maintain mitochondrial function and DNA repair. In some cases, the mitochondrial autophagy process can increase mitochondrial ROS levels, which can trigger the cell to further induce mitochondrial autophagy, thereby propagating elevated mitochondrial ROS levels through a positive feedback loop [90]. Enzymatic and non-enzymatic defense systems within the mitochondria eliminate excess ROS to protect cells from oxidative stress [91]. Non-enzymatic defense systems include flavonoids, vitamins, glutathione, SOD, superoxide reductase (SOR), catalase (CAT), glutathione peroxidase (GPX), glutathione disulfide reductase (GSR), peroxiredoxin (PRDX) and thioredoxin (TXN) [92].

As the main acetyl-lysine deacetylase within the mitochondria, SIRT3 can regulate several proteins involved in mitochondrial function and ROS production [93]. SIRT3 can regulate ROS clearance mainly by altering the acetylation of SOD2 [94, 95]. More importantly, SIRT3 directly binds and deacetylates SOD2, thereby increasing SOD2 activity and significantly influencing ROS homeostasis and autophagic flux within the mitochondria [82, 96]. As a result, increased mitochondrial ROS production is an important stimulus for the development of autophagy in several diseases. Autophagic degradation and removal of damaged oxidized proteins in response to mitochondrial oxidative stress have been reported to be beneficial for cells [97, 98]. Conversely, severe oxidative stress and increased mitochondrial ROS can activate signaling pathways that induce autophagic cell death [99], which may have some detrimental effects on cells.

### Other pathways

Studies have shown that SIRT3 uses its deacetylase activity to prevent mitochondrial damage during acute kidney injury (AKI). It can also protect the kidney from ischemia-reperfusion injury (IRI) by modulating the dynamin-related protein 1 (DRP1) pathway to induce mitochondrial autophagy [33]. SIRT3 also protects the kidney from IRI via SIRT3-glutathione S-transferase P1 (GTSP1)/c-Jun amino-terminal kinase (JNK), which inhibit autophagy and exacerbate sunitinib-induced cardiotoxicity [100]. Similarly, overexpression of SIRT3 in high glucose-stimulated human renal tubular epithelial (HK-2) cells can increase the levels of autophagy regulators. Overexpression of SIRT3 restored the dynamic balance of autophagosome/autolysosome by targeting the MTOR/ULK1 signaling pathway, and the results showed that SIRT3 effectively attenuated the cardiotoxicity of doxorubicin (DOX), providing the theoretical basis for further exploration of disseminated intravascular coagulation (DIC) [101]. In addition, SIRT3 activates autophagy, at least in part, by inhibiting the Notch-1/Hes-1 pathway. Therefore, SIRT3 may be a viable target for the treatment of diabetic nephropathy by inhibiting the Notch-1/Hes-1 signaling [34].

## Summary

Autophagy is a cellular recycling pathway that is essential for maintaining cellular integrity and intracellular homeostasis. As a result, autophagy plays an important role in the development of many diseases. SIRT3 is a mitochondrial deacetylase with diverse substrates that can be involved in various cell biological processes such as catabolism, adenosine triphosphate (ATP) production, scavenging of ROS, promotion of angiogenesis, induction of autophagy and maintenance of metabolic homeostasis. SIRT3 has a complex interaction with autophagy. SIRT3 and autophagy jointly influence the development of many diseases, for example, SIRT3 can regulate cellular autophagy through the PI3K/AKT/mTOR, SIRT3/AMPK/mTOR, SIRT3/FoxO1/FoxO3a, and SIRT3/SOD/ROS signaling pathways. However, more research is required to identify additional pathways related to the development of SIRT3 and autophagy-mediated diseases. and to identify new drug targets for SIRT3-mediated autophagy-related diseases.

## Data Availability

No datasets were generated or analysed during the current study.
